# The 53BP1 Homolog in *C. elegans* Influences DNA Repair and Promotes Apoptosis in Response to Ionizing Radiation

**DOI:** 10.1371/journal.pone.0064028

**Published:** 2013-05-08

**Authors:** Jin-Sun Ryu, Sang Jo Kang, Hyeon-Sook Koo

**Affiliations:** Department of Biochemistry, College of Life Science and Biotechnology, Yonsei University, Seoul, Republic of Korea; University of North Carolina, United States of America

## Abstract

53BP1 contributes to activation of the G2/M checkpoint downstream of ATM and MDC1 in response to ionizing radiation and promotes nonhomologous end-joining (NHEJ) in mammalian cells. In order to determine whether the cellular activities of 53BP1 are conserved in the model organism *C. elegans*, we analyzed the function of its homolog, HSR-9 in response to DNA damage. Deletion or *Mos1*-insertion in *hsr-9* did not affect the sensitivity of worms to double strand DNA breaks (DSBs), as reflected in embryonic survival and larval development. Nevertheless, the *hsr-9* mutations, as well as a *lig-4* deletion, reversed the hypersensitivity of *rad-54*-deficient worms to DSBs. In addition, oocyte chromosomal aberrations, which were increased by *rad-54* knockdown in response to DSBs, were also reduced by the *hsr-9* mutations. The *hsr-9* mutations did not prevent the cell cycle arrest induced by DSBs in mitotically proliferating germ cells. However, they attenuated apoptosis induced by DSBs, but not when CEP-1 (a p53 ortholog) was absent, suggesting that HSR-9 functions in the same pathway as CEP-1. We concluded that the 53BP1 homolog in *C. elegans* is not directly involved in cell cycle arrest in response to DSBs, but that it promotes apoptosis and also a form of NHEJ that occurs only when *rad-54* is deficient.

## Introduction

53BP1 (p53 binding protein 1) was discovered as a p53-binding protein and its primary role was initially thought to be stabilizing p53 in response to ionizing radiation [1], [2]. However, more important roles in cell cycle checkpoints and DNA repair were identified later. When double-strand DNA breaks (DSBs) are formed, ATM is activated with the help of the Mre11/Rad50/NBS1 (MRN) complex and phosphorylates histone H2AX, MDC1, and 53BP1 [3], [4]. MDC1 recruits RNF8 (RING-finger protein 8), which ubiquitinates histones H2A and H2AX, and then 53BP1 [5]–[9]. 53BP1 activates CHK2 and SMC1, and leads to activation of the G2/M and S phase checkpoint [2], [10]–[13]. 53BP1 and MDC1 were found to mediate checkpoint activation only at low doses of ionizing radiation, but were later observed to participate in maintaining G2/M arrest even at high doses of ionizing radiation [14]. Although 53BP1 is a downstream target of ATM, it can also stimulate the kinase activity of ATM [15].

In addition to its role in cell cycle arrest, 53BP1 promotes nonhomologous end-joining (NHEJ) and suppresses homologous recombination (HR) [7]. On the other hand, the upstream checkpoint protein, MDC1, mediates homologous recombination [7]. *Brca1*-deficiency (*Brca1* ^Δ11/Δ11^) induces the senescence and death of MEF cells, but this is rescued by deletion of 53BP1 [16]. It has been suggested that this rescue occurs because 53BP1 inhibits DNA-end resection by CtIP in *Brca1*-deficient cells, thus suppressing homologous recombination and permitting NHEJ. Similarly, the 53BP1 ortholog in *S. cerevisiae*, Rad9, inhibits DNA resection at DSBs and uncapped telomeres [17]. The unprotected telomeres present in TRF2 knockout cells have greater mobility in the presence of 53BP1, and this leads to telomere fusions via NHEJ [18]. Similarly, 53BP1 is required for class-switch recombination and facilitates long-range DNA end-joining in V(D)J recombination in lymphocytes [13], [19], [20].

53BP1 has a number of phosphorylation sites [21] and contains dimerization, tandem Tudor, and BRCT domains. The dimerization and BRCT domains contribute to oligomerization of 53BP1, and the BRCT domain mediates interaction with other DNA damage response proteins such as the MRN complex [15]. The oligomerized tandem Tudor domain is sufficient for recognition of DSBs and binds to di-methylated histones at H3K79 and H4K20 [22], [23]. DOT1L, an H3K79 methyltransferase, is needed to recruit 53BP1 to DSBs in human cell lines, and Dot1 plays the same role in nuclear localization of Rad9 (a 53BP1 ortholog) in *S. cerevisiae* [24]. The histone H4K20 methyltransferases, SUV4-20, SET8 (PR-SET7/8), and MMSET (WHSC1) are reported to affect 53BP1 accumulation at DSBs in human cell lines [25]–[27]. Likewise, histone methylation at H4Lys20 mediated by Set9 controls the recruitment of Crb2 (a 53BP1 ortholog) to DSBs in *S. pombe* [28], [29].

DNA damage checkpoint proteins, such as ATM, ATR, CHK1, CHK2, MRE11/RAD50, the 9-1-1 complex, and RPA, are functionally conserved in *C. elegans* [30]–[33]. In order to assess which 53BP1 activities related to DSBs are conserved in the *C. elegans* homolog HSR-9, we investigated the roles of HSR-9 in cell cycle control, DNA repair, and apoptosis after DSB formation.

## Results

### The *C. elegans* 53BP1 homolog, HSR-9 and its nuclear localization

**Figure 1:**
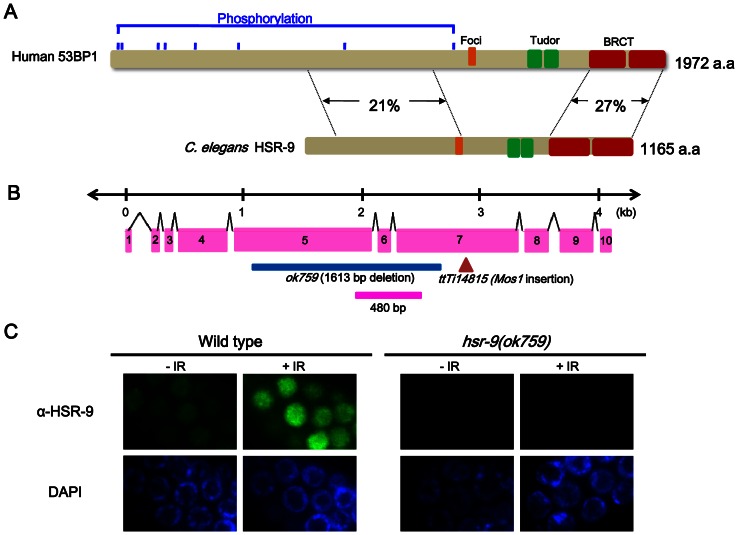
The 53BP1 homolog in *C.* *elegans*, HSR-9 protein, accumulates in the nuclei of *C. elegans* germ cells in response to γ-rays. (A) HSR-9 is the most similar of the predicted open reading frames in *C. elegans* to human 53BP1 in its C-terminal BRCT domains (27% amino acid identity) and N-terminus (21% amino acid identity). The regions controlling nuclear focus formation (Foci) and the Tudor domains are also conserved [22], [34]. (B) The deletion in *hsr-9(ok759)* is shown in blue in this representation of the gene, and the *Mos1* insertion is in brown. The polypeptide fragment used for antibody production is indicated by the pink bar. (C) HSR-9 was immuno-localized to the nuclei of wild-type germ cells in the mitotically proliferating region of gonads 3 h after γ-ray (75 Gy) treatment. Scale bar, 10 µm.

By carrying out a BLAST search of the *C. elegans* “orfeome”, we identified a protein called HSR-9 that had 27% amino acid identity with 53BP1 in the C-terminal BRCT region (*Hs* 53BP1: amino acids 1678∼1965, *Ce* HSR-9: 871∼1154), and 21% identity in the N-terminal domain (53BP1: 704∼1142, HSR-9: 111∼562) (Figure 1A). Moreover, the regions needed for nuclear focus formation (53BP1, 1251∼1271; HSR-9, 564∼582) and the tandem Tudor folds (53BP1, 1486∼1602; HSR-9, 734∼854), which bind to methylated histones, were conserved [22], [34]. To assess whether *C. elegans* HSR-9 is a functional homolog of 53BP1, we examined its intracellular location using antibodies against a polypeptide fragment of HSR-9 (496∼655 a.a.) (Figure 1B). Immuno-stained HSR-9 was observed in the nuclei of proliferating germ cells under normal conditions and the localization was much more prominent after γ-irradiation (Figure 1C). There was no signal in the *hsr-9(ok759)* germ cells either before or after γ-irradiation (Figure 1C), confirming that the antibody was specific for HSR-9. The nuclear level of HSR-9 protein also increased after UV treatment and DNA replication inhibition using hydroxyurea (Figure S1 in File S1), which suggests a role in response to various types of DNA damage.

### Mutations of *hsr-9* do not affect sensitivity to DSBs in the germ line and noncycling somatic cells

**Figure 2:**
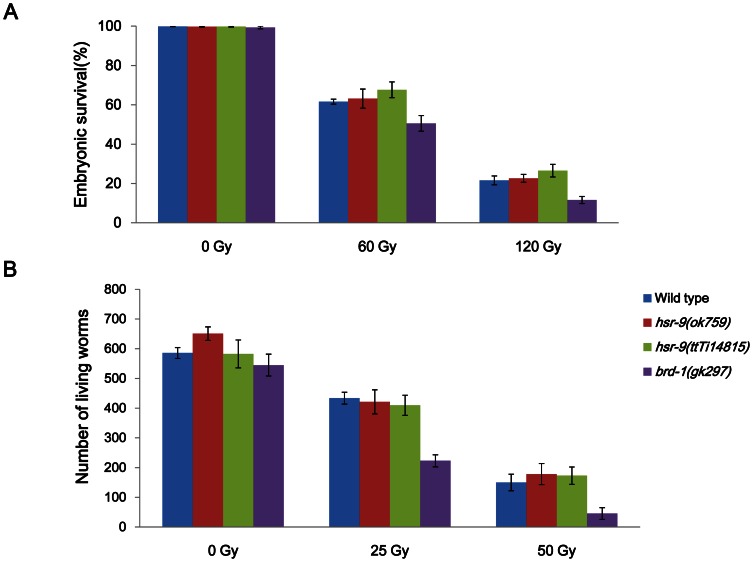
HSR-9 does not affect the survival of L4 stage germ cells or the proliferation of L1 stage germ cells. (A) Embryonic hatching was scored after exposing L4 stage worms of wild-type N2 and *hsr-9* mutants to γ-rays and collecting embryos between 24 and 48 h after irradiation. Hatching rates of wild-type N2, *hsr-9(ok759),* and *hsr-9(ttTi14815)* mutant embryos were not significantly different at 60 Gy (Student’s *t* test: N2 vs. *ok759*, *p* = 0.48; N2 vs. *ttTi14815*, *p*  = 0.13) or at 120 Gy (N2 vs. *ok759*, *p* = 0.99; N2 vs. *ttTi14815*, *p* = 0.36). (B) Five P0 worms of each strain were exposed to γ-rays (25 and 50 Gy) at the L1 stage and grown for 2 days. F1 embryos were collected over the following 24 h, and live larvae (L1-L3 stage) that hatched from the embryos were counted 24 later. *p* values between N2 and *hsr-9*(*ok759* or *ttTi14815*) at all radiation doses were larger than 0.5, except for *p* = 0.05 at 0 Gy between N2 and *hsr-9(ok759)*.

In order to test whether HSR-9 has a role in the DNA damage response as expected, we obtained an *hsr-9(ok759)* mutant with a deletion from Exon 5 to Exon 7 (Figure 1B). We also used another knockout mutant *hsr-9(ttTi14815)*, which has a *Mos1* insertion in the open reading frame (Figure 1B). The genetic backgrounds of the mutants were cleaned up by outcrossing with the wild-type N2 strain. We then irradiated L4 stage worms with γ-rays and scored the hatching of progeny embryos, to detect any effects of the *hsr-9* mutations on sensitivity to DSBs (Figure 2A). The mutation *gk297* of *brd-1* which encodes the *C. elegans* BARD1 homolog, decreased embryonic hatching after γ-irradiation, as expected from its role in HR [35]. In contrast, hatching of the *hsr-9(ok759)* deletion mutant was not significantly affected at 60 Gy (Student’s *t* test, *p* value  = 0.48) or at 120 Gy (*p* = 0.99) (Figure 2A). Nor did the *Mos1* insertion mutant, *hsr-9(ttTi14815)*, differ from the wild-type N2 strain in its sensitivity to γ-rays (at 60 Gy, *p* = 0.13; at 120 Gy, *p* = 0.36). Since *hsr-9* mutations did not significantly affect the survival of L4-stage germ cells at either the mitotically proliferating or meiotic pachytene stage, worms were instead γ-irradiated at the L1 stage (Figure 2B). In L1 survival assays, the radiation-sensitivity of only mitotically proliferating germ cells can be measured by scoring adult fertility as the number of surviving larvae in the next generation [36]. The number of living larvae was significantly reduced compared with the wild type in the positive control, *brd-1(gk297)*. However, neither of the *hsr-9* mutations, *ok759* and *ttTi14815*, significantly decreased the number of worms produced after 25 and 50 Gy irradiation (N2 vs. *ok759*, *p* = 0.05 at 0 Gy, *p*>0.5 at 25 and 50 Gy; N2 vs. *ttTi14815*, *p*>0.5 at 0, 25, and 50 Gy). Therefore, it can be concluded that the *hsr-9* mutations affect neither the survival of proliferating nor of meiotic germ cells after DSB formation.

**Figure 3:**
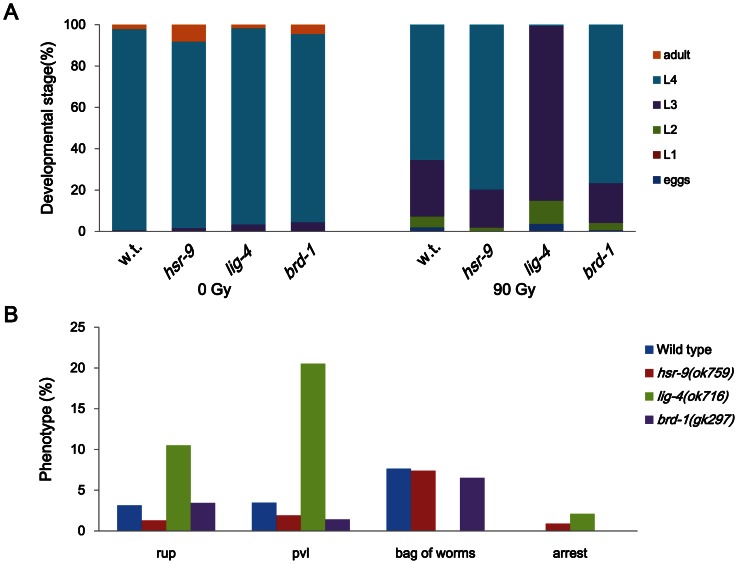
*hsr-9* deletion does not affect post-embryonic development after γ-ray treatment. Late embryos of wild-type N2, *hsr-9(ok759), lig-4(ok716),* and *brd-1(gk297)* were treated with γ-rays (90 Gy). (A) Larvae hatched from treated and untreated embryos were examined every day to estimate their growth rates, and the percentages of worms at different larval stages at 48 h after the irradiation were calculated. (B) Developmental abnormalities of treated wild-type (w.t.) and mutant worms were scored on the 3rd day of adulthood (120 h after the irradiation). Phenotypes scored were ruptured body (rup), protruding vulva (pvl), bag of worms (hatched larvae inside the mothers) and growth arrest (arrest).

Since NHEJ is important for DSB repair in non-cycling somatic cells of *C. elegans* and 53BP1 promotes NHEJ in mammalian cells [7], [37], we presumed that HSR-9, the 53BP1 homolog in *C. elegans*, would influence larval growth after DSB formation. When late embryos were treated with γ-rays (90 Gy) and cultivated to adulthood, larvae of the *lig-4(ok716)* mutant, which has a deletion in the gene for ligase 4, developed more slowly than wild-type larvae, as shown in Figure 3A. This result is very similar to the previous observations on *C. elegans* mutants deficient in NHEJ proteins such as CKU-80 (Ku80 ortholog), CKU-70 (Ku70 ortholog), and LIG-4 [38]. In contrast, *brd-1(gk297)* worms, defective in HR, did not show a significant retardation in growth rate, and neither did the *hsr-9* mutants. *lig-4(ok716)* worms developed a large number of morphological abnormalities as larvae and adults, such as ruptured body and protruding vulva, whereas wild-type N2, *brd-1(gk297)* and *hsr-9(ok759)* did not (Figure 3B). These results imply that contrary to our expectation, HSR-9 does not play a significant role in NHEJ during postembryonic development.

### *hsr-9* and *lig-4* mutations rescue the hypersensitivity to DSBs of *rad-54*-deficient germ cells

**Figure 4:**
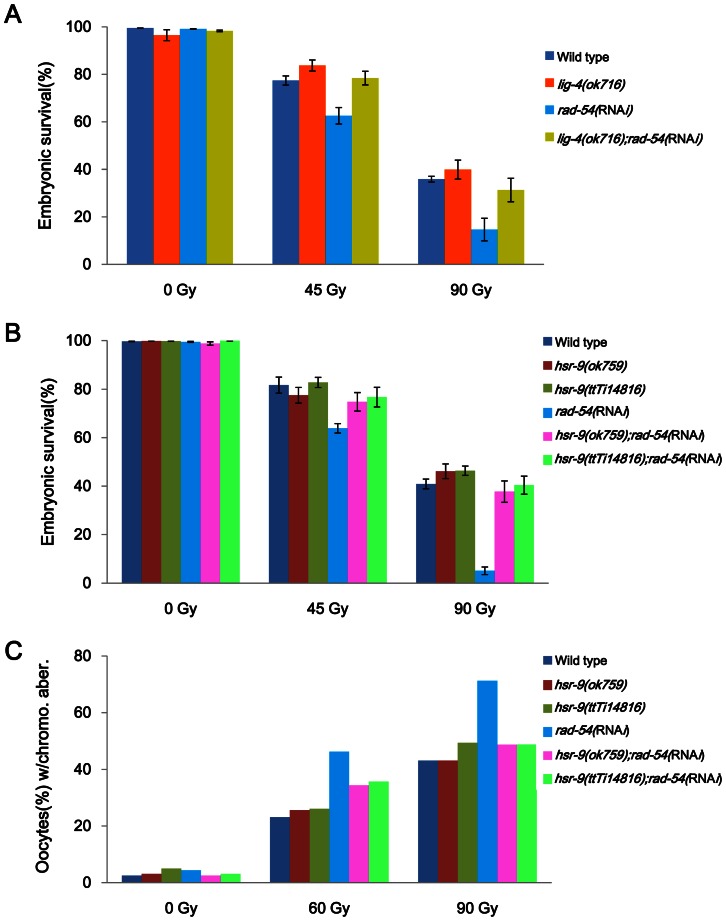
*hsr-9* mutations rescue the hypersensitivity of *rad-54*-deficient worms to γ-rays as *lig-4* mutations do. Wild-type N2, *hsr-9(ok759)*, *hsr-9(ttTi14815)*, and *lig-4(ok716)* worms were fed *E. coli* cells containing double-stranded RNA of *rad-54* from the L1 stage. (A) L4 stage worms deficient in either *lig-4* or *rad-54*, or both were treated with γ-rays (45 Gy and 90 Gy), and embryos were collected between 24 and 48 h after irradiation. The *p* values for hatching rates were: *rad-54* vs. *lig-4;rad-54*, *p* = 0.01 at 45 Gy and *p* = 0.07 at 90 Gy; *lig-4* vs. *lig-4;rad-54*, *p* = 0.24 at 45Gy and *p* = 0.26 at 90 Gy. (B) L4 stage worms deficient in either *hsr-9* or *rad-54*, or both were treated with γ-rays as in (A). The *p* values for hatching rates at 45 Gy were: *hsr-9(ok759* or *ttTi14815)* vs. *rad-54*, *p*<0.01; *rad-54* vs. *hsr-9*;*rad-54*, *p*<0.03; *hsr-9* vs. *hsr-9*;*rad-54*, *p*>0.20. The hatching rates of *hsr-9* and *hsr-9*;*rad-54* did not differ significantly at 90 Gy either (*p*>0.14). (C) L4 stage worms were irradiated with 60 and 90 Gy of γ-rays and fixed after 24 h. After staining worms with DAPI, the number of chromosomes in each oocyte was counted under a fluorescence microscopy (80 oocytes per strain). The percentage of chromosomal aberrations was calculated from the number of oocytes containing abnormal number of chromosomes (as opposed to 6 bivalent chromosomes) divided by the total number of oocytes observed.

Homologous recombination (HR) is the major DSB repair pathway in the germ cells and cycling somatic cells of *C. elegans*, whereas NHEJ is the major pathway in noncycling somatic cells [38]. Nevertheless, NHEJ influences the sensitivity of germ cells to DNA damage in particular genetic backgrounds. For example, *lig-4* mutation suppresses the hypersensitivity of *fcd-2* (a FANCD2 ortholog) mutant germ cells to interstrand DNA crosslinks (ICLs) and also suppresses the association of oocyte chromosomes seen in the double-deficiency strain of *fcd-2* and a meiotic recombination gene [39]. Therefore, we examined the possibility that *lig-4* mutation might affect the hypersensitivity to DSBs of worms defective in homologous recombination. Knockdown of the *C. elegans* ortholog of RAD54 which participates in strand invasion and branch migration during HR [38], [40]–[43], decreased embryonic survival after γ-irradiation (Figure 4), as reported previously [38]. The deletion mutation *ok716* of LIG-4, which is involved in the NHEJ pathway, expectedly rescued the embryonic lethality in *rad-54*(RNA*i*) worms after γ-irradiation (Figure 4A).

We then tested whether HSR-9, whose homolog promotes NHEJ in mammalian cells [7], [16], [18], also influences DSB repair in worms deficient in RAD-54. Indeed, both *hsr-9* mutations, *ok759* and *ttTi14815*, rescued the embryonic lethality of *rad-54*(RNA*i*) at 45 and 90 Gy (Figure 4B), as did the *lig-4(ok716)* mutation. Rescue by the *hsr-9* mutations was also observed with respect to oocyte chromosomal aberrations (defined by abnormal numbers of chromosomes) after γ-ray treatment (60 and 90 Gy) (Figure 4C): the percentage of abnormal oocytes was about 2 fold greater (2.0 fold at 60 Gy, 1.7 fold at 90 Gy) in *rad-54*(RNA*i*) than in the wild type, whereas the increase by *rad-54* knockdown was significantly less in the *hsr-9* mutant backgrounds (<1.4 fold at 60 Gy, <1.1 fold at 90 Gy).

### *hsr-9* mutations do not influence normal cell cycle arrest in response to DSBs

**Figure 5:**
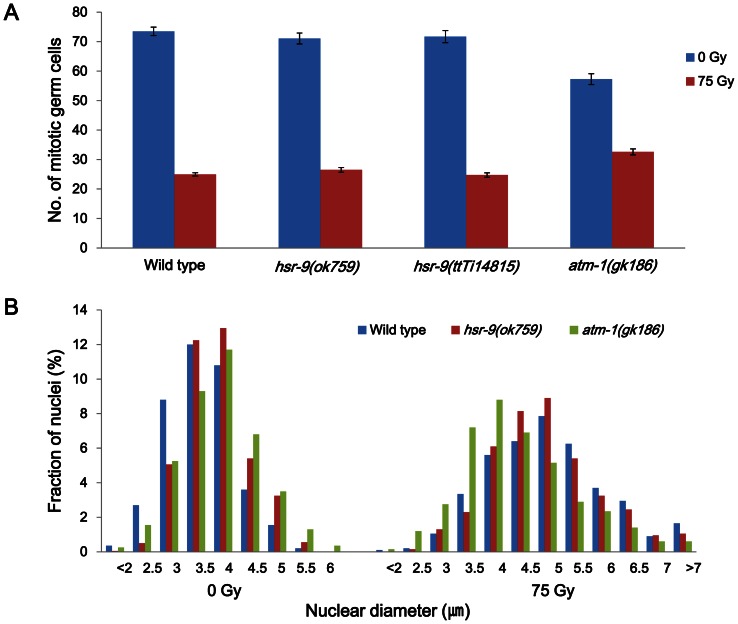
Cell cycle arrest in response to γ-rays is normal in the hsr-9(ok7599) mutants. Wild-type N2, *hsr-9(ok759)*, *hsr-9(ttTi14815)*, and *atm-1(gk186)* worms were treated with 75 Gy of γ-irradiation as L4 stage larvae. Twelve hours later, gonads were isolated and stained with DAPI. (A) Germ cells in the mitotically proliferating regions of the gonads (within 75 µm of the distal tip cell) were counted under a fluorescence microscope (30 gonad arms per data point). (B) The diameters of 40 nuclei in the mitotically proliferating region of each gonad arm were measured (number of nuclei  = 800 for each strain before and after irradiation) for wild-type N2, *hsr-9(ok759)*, and *atm-1(gk186)* worms. The percentages of the total nuclei of different diameters are plotted. Very small (<2 µm) and very large (>7 µm) nuclei were grouped together.

To detect a role of HSR-9 in the DNA damage checkpoint, gonads were stained with DAPI at 12 h after γ-ray treatment (75 Gy) and the number of nuclei in the mitotic region of each gonad arm was counted. Numbers of germ cells were decreased to the same extent in the two *hsr-9* mutants as in wild-type N2 (N2, Δn = 49±2(SEM); *hsr-9(ok759)*, Δn = 45±2; *hsr-9(ttTi14816)*, Δn = 47±2; *p*>0.5 between N2 and *hsr-9* mutants), whereas the reduction was less in *atm-1(ok186)* (N2, Δn = 49±2(SEM); *atm-1(gk186)*, Δn = 25±2; *p*<0.03) (Figure 5A). This suggests that unlike ATM-1, HSR-9 is not significantly involved in cell cycle arrest. We also quantified the extent of cell cycle arrest by measuring the diameters of germ cell nuclei in the mitotically proliferating regions of gonads. Germ cells normally enlarge after γ-irradiation due to G2 arrest and subsequent cell growth [44]–[46]. Before γ-ray treatment, the nuclear diameters of the *hsr-9* and *atm-1* mutants were slightly larger than those of wild type N2 (N2, average nuclear diameter (d) = 3.60±0.02(SEM)µm; *hsr-9(ok759)*, 3.87±0.02 µm; *atm-1(gk186)*, 3.92±0.02 µm). As expected, after γ-ray treatment, their average values (d) increased in all three strains (N2, 5.01±0.04(SEM) µm; *hsr-9(ok759)*, 4.94±0.04 µm; *atm-1(gk186)*, 4.42±0.04 µm). However, the distribution of nuclear sizes in the *hsr-9(ok759)* worms was much closer to that in wild-type worms than to that in *atm-1(ok186)* worms, and consequently the same was true for averages of nuclear diameters (Figure 5B). This result agrees with that in Figure 5A, supporting the view that HSR-9 does not play a significant role in cell cycle arrest after DSB formation, at least not one as important as that of ATM-1.

### *hsr-9* mutations attenuate apoptosis induced by DSBs

**Figure 6:**
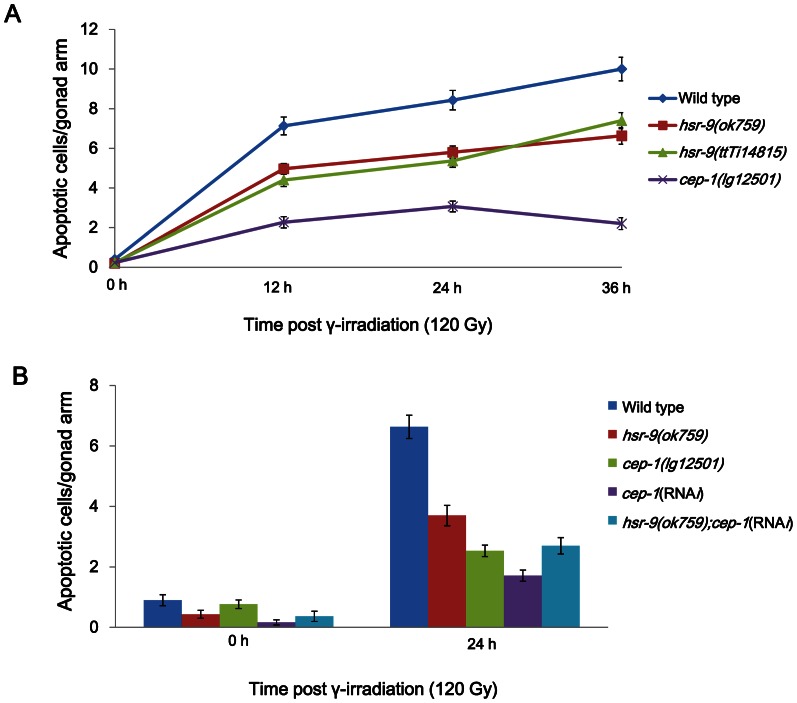
*hsr-9* mutations attenuate apoptosis induced by γ-rays. (A) Wild type, *hsr-9(ok759), hsr-9(ttTi14815)*, and *cep-1(lg12501)* L4 stage worms were irradiated with γ-rays (120 Gy), and stained with SYTO-12 dye at 0, 12, 24, and 36 h after γ-ray treatment. Intensely stained pachytene stage apoptotic cells were scored in each gonad arm. (B) Wild type and *hsr-9(ok759)* worms were fed *E. coli* cells containing double-stranded RNA of *cep-1* from the L1 stage. Wild type, *hsr-9(ok759), cep-1(lg12501)*, *cep-1(*RNA*i)*, and *hsr-9(ok759);cep-1(*RNA*i)* L4 stage worms were treated with γ-rays (120 Gy), and apoptotic germ cells were counted as in (A).

Although we did not detect a role of HSR-9 in cell cycle arrest in response to DSBs, we examined whether the deletion mutation affects apoptosis induced by DSBs. In the *C. elegans* germline, more than half of the germ cells spontaneously undergo apoptosis at the pachytene stage, and this can be detected by either observing cell corpses under Nomarski optics or staining for condensed chromosomes [47]. L4 stage hermaphrodites were treated with γ-rays (120 Gy), and dying germ cells at the pachytene stage were scored at 12 h intervals after staining with SYTO-12. *hsr-9(ok759)* and *hsr-9(ttTi14815)* mutants contained fewer apoptotic cells than wild-type worms (Figure 6A). However, the reductions of apoptotic cell numbers in *hsr-9(ok759)* and *hsr-9(ttTi14815)* were less than in *cep-1(lg12501)*, which has a deletion in the gene coding for a P53 ortholog [48], [49]. In order to determine whether HSR-9 influences apoptosis via the same pathway as CEP-1, we measured apoptotic cells of *hsr-9(ok759);cep-1(*RNA*i)* after γ-ray treatment (120 Gy, 24 h later). The apoptotic cell number in this strain was similar to that in *cep-1(lg12501)* and slightly higher than in *cep-1(*RNA*i)* (*hsr-9(ok759);cep-1(*RNA*i)* vs. *cep-1(lg12501)*, *p* = 0.62; *hsr-9(ok759);cep-1(*RNA*i)* vs. *cep-1(*RNA*i) p*<0.01) (Figure 6B). The fact that the reductions in apoptotic cell numbers in the two single deficiency strains did not add up to the reduction in the double deficiency strain, suggests that HSR-9 functions in the same pathway as CEP-1. To confirm this, transcripts of *egl-1*, a cell-death activator downstream of *cep-1* [50], were measured by gene-specific real-time PCR after reverse transcribing total RNA (Figure S2 in File S1). The transcripts were significantly lower in *hsr-9(ok759)* worms than in wild-type worms (*p* = 0.01), but not as low as in *cep-1(lg12501)* worms. The reduced level of *egl-1* transcripts in *hsr-9(ok759)* is likely to have contributed to the reduction in apoptosis.

## Discussion

53BP1-deficient mice are growth-retarded, immuno-deficient, radiation-sensitive, and cancer prone [51]. In contrast, we found that *C. elegans* mutants of the 53BP1 homolog, HSR-9, were not hypersensitive to DSBs as measured by embryonic survival and larval growth after γ-ray treatment (Figures 1 and 2). However, the *hsr-9* mutations unexpectedly reversed the hypersensitivity to DSBs in *rad-54*-deficient worms, suggesting that it has a role in DSB repair (Figure 4). The observation is very similar to the finding that the phenotypes of BRCA1-deficient (*Brca1* ^Δ11/Δ11^) mice, such as embryonic lethality, senescence, and cell death, are rescued by 53BP1 knockout [16]. The suppression of the BRCA1-deficiency phenotypes by 53BP1 mutation has been explained by the mechanism that 53BP1 inhibits end-resection at DSBs, thereby shifting the DSB repair towards NHEJ rather than HR [37]. Thus, although HR is compromised in BRCA1-deficient cells and leads to error-prone repair by NHEJ, the absence of 53BP1 allows DSBs to be repaired by error-free HR. To test whether this genetic relationship was conserved in *C. elegans*, we generated a double mutant of *brc-1* (BRCA1 homolog) and *hsr-9*. However, the *brc-1(tm1145);hsr-9(ok759)* mutant was as hypersensitive to DSBs as *brc-1(tm1145)*, in contrast to the mammalian situation (Figure S3 in File S1, *brc-1;hsr-9* vs. *brc-1*, *p*>0.3). This difference is probably related to the fact that the CTIP homolog in *C. elegans*, COM-1, is not required for RAD-51 loading at exogenously-induced DSBs, but is essential at meiotic DSBs generated by SPO-11 [52]. Therefore, *C. elegans* CTIP does not appear contribute to the processing of exogenously-induced DSBs and so is unlikely to interact with BRC-1 during end-resection. Instead, the *C. elegans* homolog of RAD54, which is involved in strand invasion and branch migration during homologous recombination, interacts with the 53BP1 homolog, HSR-9 (Figures 4B and 4C). A deletion mutation of *lig-4*, which is involved in NHEJ pathway, also rescued the embryonic lethality due to *rad-54* knockdown after γ-ray treatment (Figure 4A). The fact that both HSR-9 and LIG-4 are involved in similar genetic interactions with RAD-54 suggests that HSR-9 probably promotes NHEJ in the context of the HR defect associated with RAD-54 deficiency. Although it is clear that *hsr-9* mutation rescues *rad-54* knockdown phenotypes, the mechanism responsible is not evident. One possibility is that RAD-54 interacts with the MRE-11 or EXO-1 nucleases during the end-resection of DSBs, as BRCA1 does with CTIP in mammalian cells. Another possibility is that chromatin modulation by RAD-54, a SNF2 family protein, is normally counterbalanced by binding of HSR-9 to di-methylated histones near DSBs [22], [23]: in the absence of RAD-54 at DSBs, abnormal chromatin structures may be formed as a result of HSR-9 activity, and HR cannot proceed.

The pan-nuclear level of HSR-9 was significantly increased in germ cells after DSB formation (Figure 1C), but there were no clear foci of HSR-9 in contrast to the case of mammalian 53BP1. Similarly, *C. elegans* ATM-1 did not form nuclear foci unlike its mammalian homolog [53], implying that the homologs of mammalian proteins that form nuclear foci in response to DNA damage do not always form foci in worms. 53BP1 has several S/TQ sites that can be phosphorylated by ionizing or UV radiation, which is consistent with the idea that ATM and ATR catalyze its phosphorylation [21]. Indeed, we observed that nuclear HSR-9 increased after inhibiting DNA replication with hydroxyurea and after exposure to UV–radiation (Figure S1 in File S1). Despite the nuclear accumulation of HSR-9, *hsr-9* mutations did not affect the arrest of germ cell proliferation induced by DSBs, whereas the *atm-1* mutation did partially prevent the arrest (Figure 5). Thus, HSR-9 does not seem to participate in checkpoint activation, unlike its homolog in mammalian cells. One reason for this difference could be the fact that the *C. elegans* homolog of CHK2, a downstream target of 53BP1, does not play a significant role in the response to DSBs [54], [55]. Another reason could be the high dose of γ-rays that we used, because a need for 53BP1 in checkpoint activation was observed only at low doses of ionizing radiation [2], [12].

Previously, *hsr-9* knockdown was found to result in reduced progeny survival, defective cell cycle arrest, and attenuated apoptosis in response to DSBs in a high-throughput protein interaction analysis of DNA damage responses in *C. elegans* [56]. In contrast to these effects, we did not observe any effects of *hsr-9* mutations on progeny survival or cell cycle arrest. Nevertheless, *hsr-9* mutations did attenuate germline apoptosis after DSB formation (Figure 6), like *hsr-9* knockdown in the previous report [56]. Double deficiency for *hsr-9* and *cep-1* (the p53 ortholog in *C. elegans*) did not further decrease apoptotic cell numbers in the germline compared to single deficiency for *cep-1*, suggesting that HSR-9 promotes apoptosis induced by DSBs via the same pathway as CEP-1. The fact that *egl-1* mRNA expression, which is induced by CEP-1, is decreased by *hsr-9* mutation further confirms that the two proteins function in the same pathway (Figure S2 in File S1). HSR-9 probably mediates the activation of CEP-1 in response to DSBs, as 53BP1 contributes to the accumulation of p53 in mammalian cells [2]. It is intriguing that RAD-54 and MRE-11, acting in the homologous recombination pathway, promote apoptosis in response to UV-C radiation in *C. elegans* [41], as HSR-9 does in response to DSBs.

We conclude that HSR-9 mediates apoptosis induced by DSBs in the same pathway as CEP-1. However, there is no clear evidence that HSR-9 functions in cell cycle arrest. Similarly, normal cell cycle arrest was observed in response to DSBs after knocking down CEP-1 [49]. In spite of the fact that HSR-9 appears not to function in DSB repair in the wild type genetic background, HSR-9 mutations reverse the hypersensitivity of RAD-54-deficient cells to DSBs like LIG-4 deficiency. This suggests that HSR-9 can shift the choice of DSB repair pathway from HR to NHEJ, just as 53BP1 promotes NHEJ in BRCA1-deficient cells. Therefore, the roles of 53BP1 in mammalian cells, mediating apoptosis and shunting DSB repair to NHEJ, are to a degree conserved in the 53BP1 ortholog of *C. elegans* [16], [37].

## Materials and Methods

### C. elegans strains

The standard wild-type Bristol N2 strain, as well as the *hsr-9(ok759), atm-1(gk186*), *brd-1(gk297)*, *lig-4(ok716)*, *cep-1(lg12501)*, *and brc-1(tm1145)* mutants were obtained from the *Caenorhabditis* Genetics Center (Minneapolis, MN, USA). We obtained a validated *hsr-9(ttTi14815)* strain (with a *Mos1* transposon inserted in the 7th exon of *hsr-9*) from Drs. Laurent Ségalat (Université Claude Bernard Lyon 1, France) and Maïté Carre-Pierrat (Plateforme “Biologie de *Caenorhabditis elegans*”, France). To generate uniform genetic backgrounds, *hsr-9(ok759), hsr-9(ttTi14815), brd-1(gk297)*, *brc-1(tm1145)*, and *lig-4(ok716)* were outcrossed with wild type N2 worms 4, 3, 3, 6, and 2 times, respectively. The double mutant *brc-1(tm1145);hsr-9(ok759)* was generated by crossing *brc-1(tm1145)* with *hsr-9(ok759)*. The *atm-1(gk186)* mutant had been previously outcrossed 6 times [53]. All strains were maintained by feeding them *E. coli* OP50-1 cells at 20°C. To confirm that the *Mos1* transposon inserted in *hsr-9(ttTi14815)* was not spontaneously excised, the genomic DNA next to the inserted transposon was amplified from *hsr-9(ttTi14815)* (Figure S4 in File S1). When the genomic DNA fragment was analyzed by agarose gel electrophoresis, the *Mos1* insertion was found to be present.

### Bacteria-mediated RNA*i*

*E. coli HT115*(DE3) cells harboring a plasmid containing the cDNA of *cep-1* and *rad-54* were obtained from the *C. elegans* RNA*i* v1.1 feeding library (Open Biosystems) and Ahringer’s RNA*i* feeding library, respectively. The *E. coli* cells were cultured in liquid LB medium containing ampicillin (50 µg/me) and tetracycline (5 µg/me) for 12 h at 37°C. Aliquots of the cultures were spread on NGM plates containing 1 mM IPTG and carbenicillin (25 µg/me), and left at room temperature for 24 h. For *rad-54* RNA*i* and *cep-1* RNA*i*, *C. elegans* worms were grown on the plates from the L1 stage.

### L4-stage gem cell survival and oocyte chromosomal aberration after γ-ray treatment

Twenty wild type, *hsr-9(ok759)*, *hsr-9(ttTi14815), brd-1(gk297), lig-4(ok716)*, *rad-54(*RNA*i), hsr-9*;*rad-54(*RNA*i*), and *lig-4(ok716)*;*rad-54(*RNA*i*) worms at the L4 stage were exposed to 60 Gy and 120 Gy (or 45 Gy and 90 Gy) of γ-rays using a 137Cs source (IBL 437C, CIS Biointernational). Embryos were collected between 24 and 48 h after irradiation, and their hatching was scored 24 h later. To score oocytes containing abnormal numbers of chromosomes, worms were fixed 24 h after irradiation (60 and 90 Gy), stained with DAPI, and observed with a fluorescence microscope (DMRHC, Leica).

### Survival of L1-stage germ cells after γ-ray treatment

The survival of L1-stage germ cells was measured according to the protocol of Craig et al. [36] with slight modification. Wild type, *hsr-9(ok759), hsr-9(ttTi14815)* and *brd-1(gk297)* worms at the L1 stage were treated with 25 Gy and 50 Gy of γ-rays. After 48 h, five worms of the P0 generation were plated onto each of 3 NGM plates containing *E. coli* OP50-1 lawn, and embryos were collected for 24 h. The number of living worms (F1 generation) was counted 24 h later.

### Measurement of developmental abnormalities after γ-ray treatment

Twenty adult worms were allowed to lay eggs for 1 h. After further incubation for 4 h, late-stage embryos were irradiated with γ-rays (90 Gy). Hatched worms were examined with a stereomicroscope once per day to estimate their growth rates until the control (untreated wild-type) worms reached the L4 stage. Developmental abnormalities were scored when the controls reached the 3rd adult day. Phenotypes scored were ruptured body (rup), protruding vulva (pvl), bag of worms (hatched larvae inside the mothers) and growth arrest (arrest).

### Assessment of cell cycle arrest after γ-ray treatment

To measure the effect of γ-rays on cell cycle arrest, wild type, *hsr-9(ok759), hsr-9(ttTi14815),* and *atm-1(gk186)* larvae were treated at the L4 stage with γ-rays (75 Gy). After 12 h, their gonads were dissected, stained with DAPI, and observed with a fluorescence microscope (DMRHC, Leica). Cell cycle stages were determined by two methods. First, we counted the number of nuclei within 75 µmof the distal tip cells of each gonad, that is, in the mitotically proliferating region. Second, we measured the diameters of about 40 nuclei in the mitotically proliferating region of each gonad.

### SYTO-12 staining for apoptotic germ cells

To identify apoptotic cells induced by double-strand DNA breaks, wild type, *hsr-9(ok759), hsr-9(ttTi14815), cep-1(lg12501)*, and *hsr-9(ok759);cep-1(*RNA*i)* L4 stage worms were irradiated with γ-rays (120 Gy). Worms were harvested at 0, 12, 24, and 36 h after γ-ray treatment, and incubated in 33 µM SYTO-12 for 2 h at 25°C. After 3 washes in 1× PTw, they were allowed to recover for 1 h on NGM plates with bacterial lawn, and then mounted on glass slides with agar pads. Apoptotic cells at the pachytene germ stage were observed with a fluorescence microscope.

### Preparation of anti-HSR-9 antibody

Total RNA was isolated from mixed-stage *C. elegans* worms using an easy-BLUE Total RNA Extraction kit (Intron Biotechnology). A cDNA pool was synthesized from the total RNA using M-MLV reverse transcriptase (Intron Biotechnology) and oligo(dT) primer, and the full-length cDNA of HSR-9 was amplified by gene-specific PCR. PCR was also used to amplify a cDNA fragment (nucleotides 1486∼1965) of the open reading frame, which was cloned into pGEM-T vector (Promega). The forward and reverse primers were 5′-GGATCCCGTAGATCTACAAGGGCTAAG and 5′-GAATTCCGAAGTTCCTACAGACGATAC, respectively. The recombinant plasmid DNA was digested at the *Bam*HI and *Eco*RI sites (underlined), and the cDNA product was recloned into pGEX4T-1 vector. The resulting plasmid pGEX4T-1/HSR-9 was transferred into *E. coli* BL21(DE3) cells, and protein expression was induced by incubation with 1 mM IPTG for 4 h at 37°C. The fusion protein was isolated and used to raise antibodies in SD rats.

### Immunostaining of gonads

The heads of adult *C. elegans* worms were cut open, and the gonads and intestines pushed out. These were fixed in 3% paraformaldehyde and 0.1 M K_2_HPO_4_ (pH 7.2) for 1 h at room temperature. They were then washed 5 times with 1× PTw and placed in methanol at −20°C overnight for post-fixation. After 5 washes in 1× PTw, the samples were left at room temperature for 1 h in blocking solution (a mixture of equal volumes of goat serum (Gibco-BRL) and 1× PTw). The fixed specimens were reacted with HSR-9 antiserum diluted in blocking solution (1∶50) at 4°C overnight. After five washes in 1× PTw, specimens were incubated with FITC-conjugated goat anti-rat immunoglobulin (1∶1000 dilution, Molecular Probes) at room temperature for 1 h. They were washed 3 times, stained with DAPI for 15 min, and washed twice with 1× PTw. They were then placed on glass slides coated with 2% agarose, followed by addition of antifade reagent (Invitrogen), and observed with a fluorescence microscope (DMRHC, Leica).

### Quantitative RT-PCR of *egl-1* mRNA

Adult wild type, *hsr-9(ok759),* and *cep-1(lg12501)* worms were irradiated with 120 Gy of γ-rays and incubated for 6 h. Total RNA was isolated using an easy-BLUE Total RNA Extraction kit (Intron Biotechnology). Total RNA (2 µg) was reverse-transcribed using M-MLV reverse transcriptase (Intron Biotechnology) and oligo(dT) primer. Relative amounts of *egl-1* cDNA were measured by real-time PCR using IQ SYBR Green Supermix (BIORAD) in a CFX96 Detection System (BIORAD). Primer pairs were designed as suggested by Hofmann et al [30], and γ-tubulin was used as a standard to calculate the relative abundance of *egl-1* mRNA. The primer pairs were for *egl-1*, 5′-CAGGACTTCTCCTCGTGTGAAGATTC and 5′-GAAGTCATCGCACATTGCTGCTA, and for γ-tubulin, 5′-AAGATCTATTGTTCTACCAGGC and 5′-CTTGAA CTTCTTGTCCTTGAC.

### Identification of *Mos1* insertion by PCR amplification of genomic DNA

To detect the *Mos1* insertion in the 7th exon of the *hsr-9* gene, single wild type and *hsr-9(ttTi14815)* worms were lysed, and a genomic DNA fragment containing the insertion site was amplified. PCR was carried out in a 2720 Thermal Cycler (Applied Biosystems) using primers, 5′-TCTCCTGCTGCTAAGAATCG and 5′-ACGAGTAGCCTCCGATATTGT. Amplified DNA fragments were electrophoresed on a 1% agarose gel, stained with ethidium bromide, and visualized with a UV transilluminator.

## Supporting Information

File S1 **Figure S1, HSR-9 protein accumulates in the nuclei of** ***C. elegans*** **germ cells in response to various types of DNA damage.** (A) HSR-9 was immuno-localized to the nuclei of wild-type germ cells in the mitotically proliferating region of gonads in response to hydroxyurea (HU, 60 mM) treatment for 16 h. (B) Nuclear levels of HSR-9 at 3 h after UV (150 J/m^2^) irradiation. Scale bar, 10 µm. **Figure S2,** ***hsr-9*** **deletion decreases** ***egl-1*** **mRNA expression after γ-ray treatment.** Adult wild type, *hsr-9(ok759),* and *cep-1(lg12501)* worms were irradiated with 120 Gy of γ-rays, and total RNA was isolated 6 h later. cDNA pools were prepared by reverse transcription, and relative amounts of *egl-1* cDNA were measured by real-time PCR. **Figure S3,** ***hsr-9*** **mutation cannot relieve the hypersensitivity of** ***brc-1*** **mutants to γ-rays.** Wild-type N2, *hsr-9(ok759), brc-1(tm1145),* and *brc-1(tm1145);hsr-9(ok759)* worms were irradiated with γ-rays (45 Gy and 90 Gy) at the L4 stage. Embryos were collected between 24 and 48 h after the irradiation, and their hatching was scored 24 h later. *brc-1(tm1145)* mutation but not *hsr-9(ok759)* resulted in hypersensitivity to DSBs. The double mutant was very similar to the single *brc-1* mutant in its sensitivity to DSBs (at both 45 Gy and 90 Gy, *brc-1* vs. *brc-1;hsr-9*, *p*>0.3). **Figure S4, The presence of** ***Mos1*** **insertion in the** ***hsr-9(ttTi14815)*** **mutant is confirmed by PCR amplification of a genomic DNA fragment.** Single worms were picked and lysed, followed by PCR amplification of the genomic DNA fragment flanking the insertion site. A 0.75 kb DNA fragment was produced from wild-type worms, and a much longer DNA fragment (2.0 kb) from the *hsr-9(ttTi14815)* mutant.
(PDF)
