# Supplementary figures and images for: The 53BP1 Homolog in C. elegans Influences DNA Repair and Promotes Apoptosis in Response to Ionizing Radiation
Source: PLoS One. 2013 May 8;8(5):e64028. doi: 10.1371/journal.pone.0064028 (PMC3648578; doi:10.1371/journal.pone.0064028)

**A**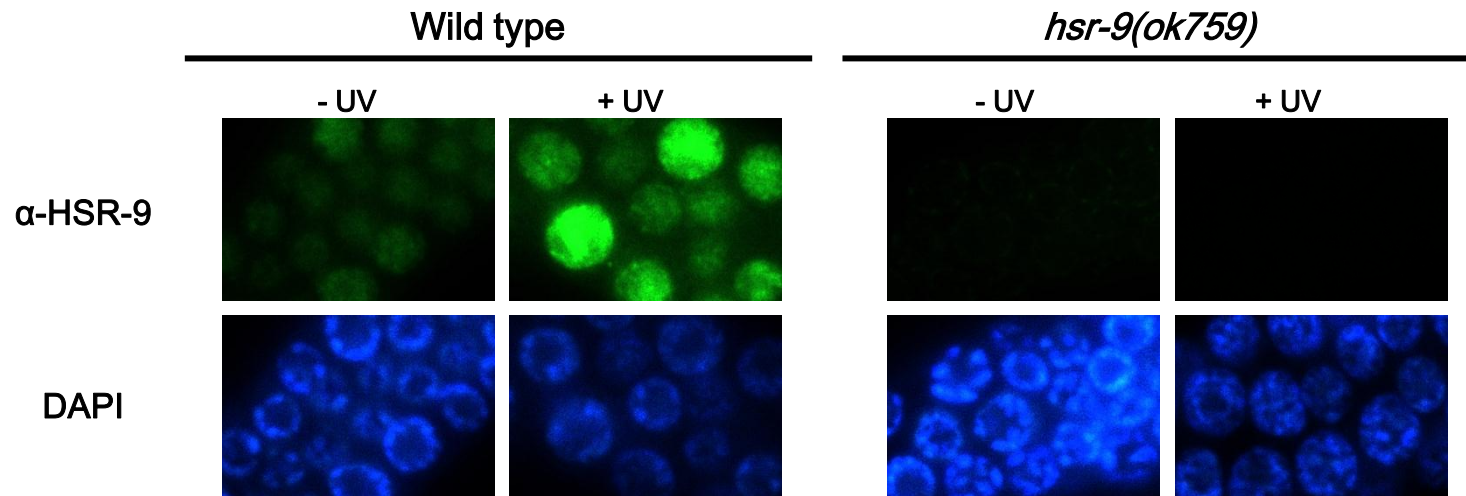**B**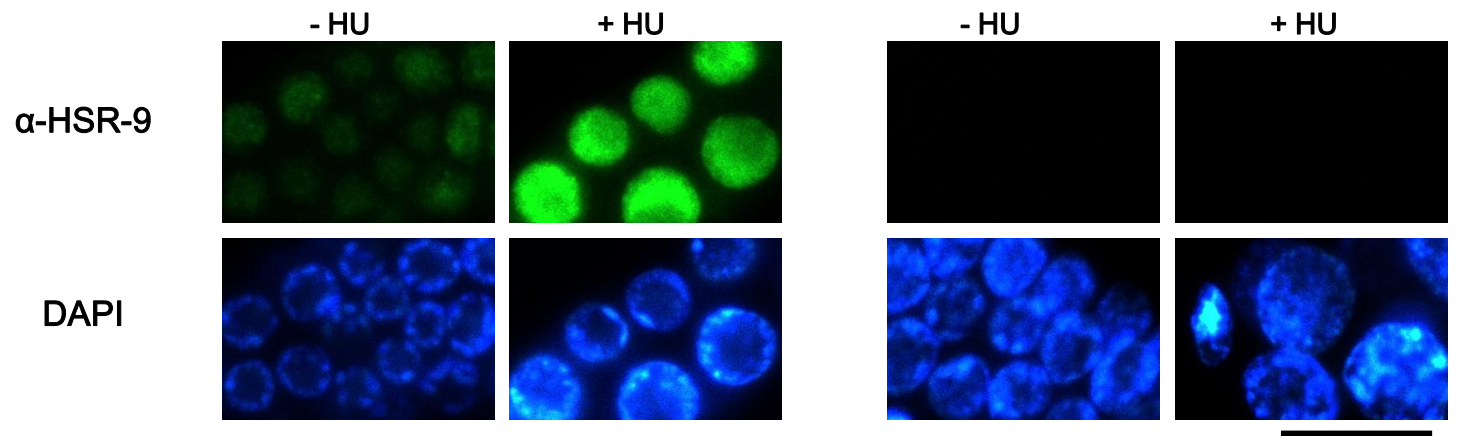**Figure S1**

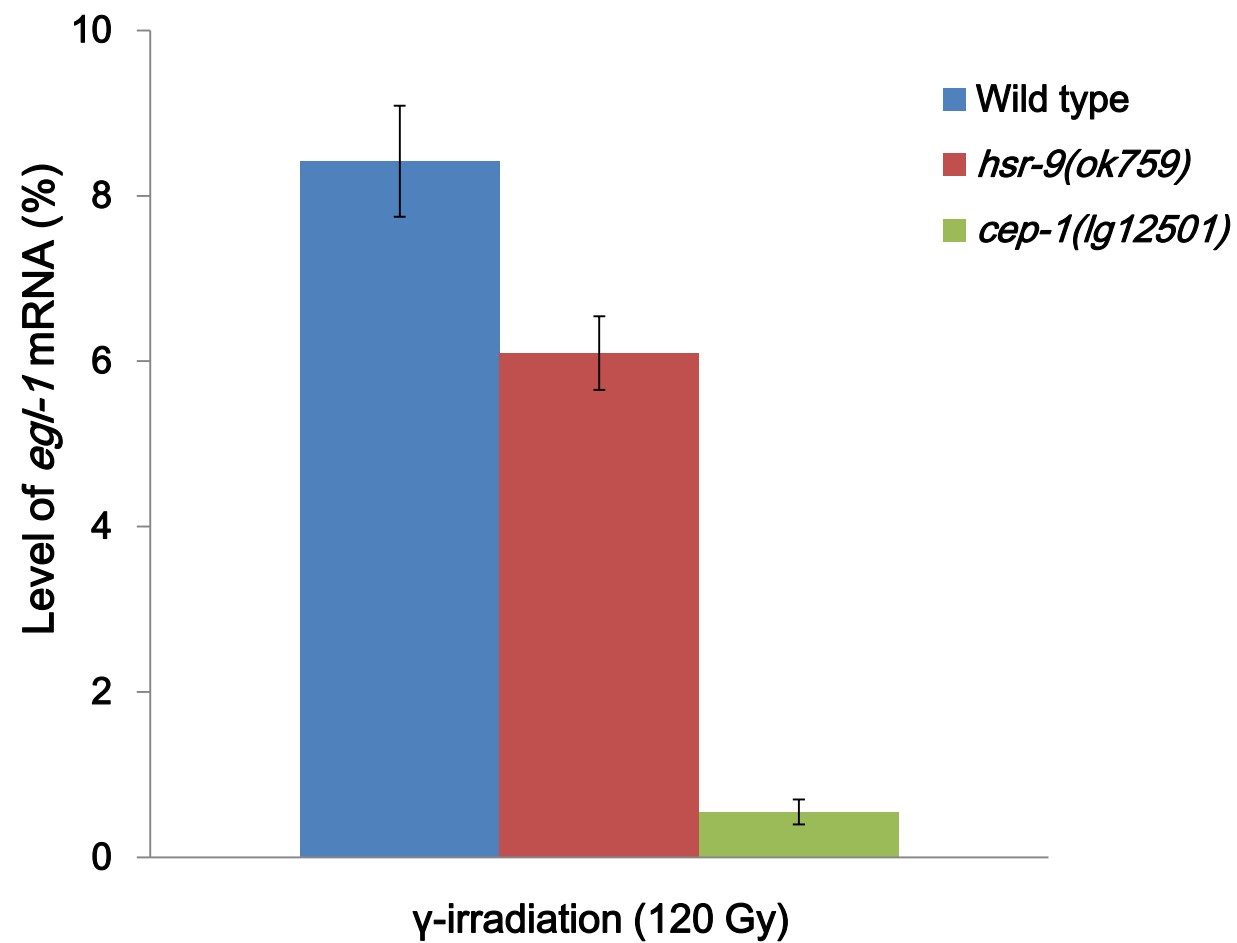

Figure S2

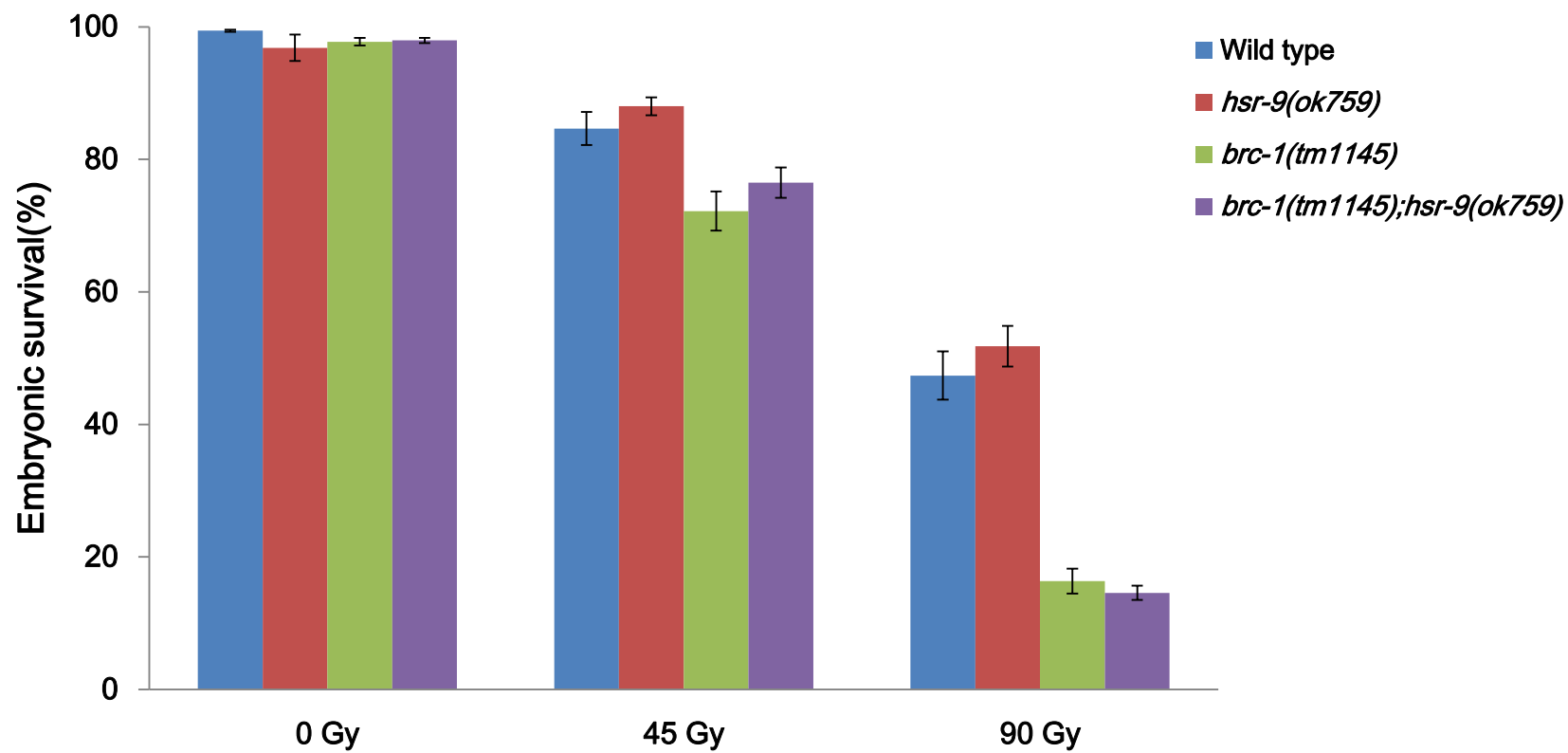

Figure S3

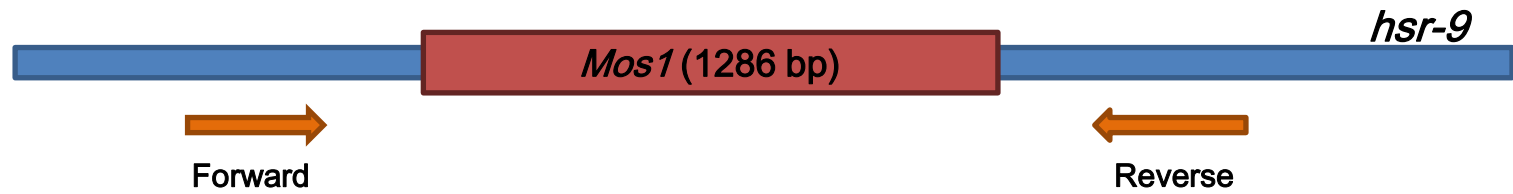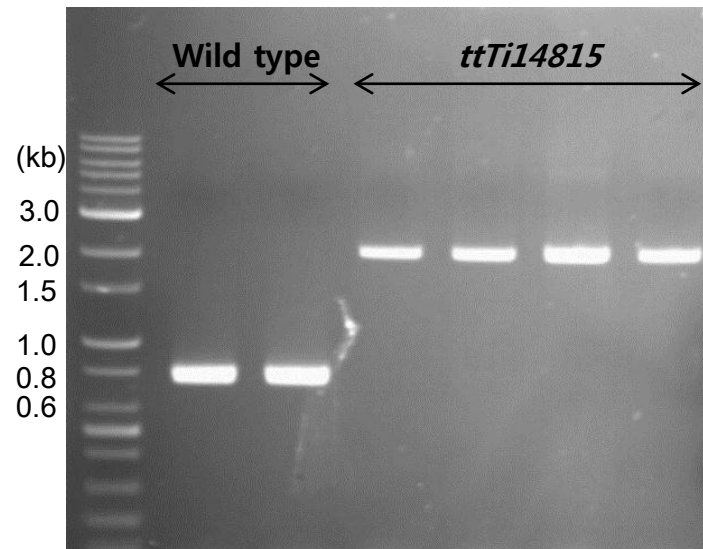

Figure S4

Supplement: File S1 — Figure S1, HSR-9 protein accumulates in the nuclei of C. elegans germ cells in response to various types of DNA damage. (A) HSR-9 was immuno-localized to the nuclei of wild-type germ cells in the mitotically proliferating region of gonads in response to hydroxyurea (HU, 60 mM) treatment for 16 h. (B) Nuclear levels of HSR-9 at 3 h after UV (150 J/m2) irradiation. Scale bar, 10 µm. Figure S2, hsr-9 deletion decreases egl-1 mRNA expression after γ-ray treatment. Adult wild type, hsr-9(ok759), and cep-1(lg12501) worms were irradiated with 120 Gy of γ-rays, and total RNA was isolated 6 h later. cDNA pools were prepared by reverse transcription, and relative amounts of egl-1 cDNA were measured by real-time PCR. Figure S3, hsr-9 mutation cannot relieve the hypersensitivity of brc-1 mutants to γ-rays. Wild-type N2, hsr-9(ok759), brc-1(tm1145), and brc-1(tm1145);hsr-9(ok759) worms were irradiated with γ-rays (45 Gy and 90 Gy) at the L4 stage. Embryos were collected between 24 and 48 h after the irradiation, and their hatching was scored 24 h later. brc-1(tm1145) mutation but not hsr-9(ok759) resulted in hypersensitivity to DSBs. The double mutant was very similar to the single brc-1 mutant in its sensitivity to DSBs (at both 45 Gy and 90 Gy, brc-1 vs. brc-1;hsr-9, p>0.3). Figure S4, The presence of Mos1 insertion in the hsr-9(ttTi14815) mutant is confirmed by PCR amplification of a genomic DNA fragment. Single worms were picked and lysed, followed by PCR amplification of the genomic DNA fragment flanking the insertion site. A 0.75 kb DNA fragment was produced from wild-type worms, and a much longer DNA fragment (2.0 kb) from the hsr-9(ttTi14815) mutant. (PDF) [file pone.0064028.s001.pdf]
